# Supplementary material for: A Sulfur-Bridging Sulfonate-Modified Zinc(II) Phthalocyanine Nanoliposome Possessing Hybrid Type I and Type II Photoreactions with Efficient Photodynamic Anticancer Effects
Source: Molecules. 2023 Feb 28;28(5):2250. doi: 10.3390/molecules28052250 (PMC10005636; doi:10.3390/molecules28052250)
Supplement: Supplementary file 1 [file molecules-28-02250-s001.zip › molecules-2224526-supplementary.pdf]

Supporting Information

A Sulfur-Bridging Sulfonate-Modified Zinc(II) Phthalocyanine Nanoliposome Possessing Hybrid Type I and Type II Photoreactions with Efficient Photodynamic Anticancer Effects

Zixuan Chen[†1], Yuan-Yuan Zhao[†1], Li Li[1], Ziqing Li[1], Shuwen Fu[1], Yihui Xu[1], Bi-Yuan Zheng[1], Meirong Ke[1], Xingshu Li [*1]and Jian-Dong Huang[*1]

[1]College of Chemistry, State Key Laboratory of Photocatalysis on Energy and Environment, Fujian Provincial Key Laboratory of Cancer Metastasis Chemoprevention and Chemotherapy, Fuzhou University, Fuzhou 350108, China

[*] Corresponding author. E-mail address: xingshuli@fzu.edu.cn; jdhuang@fzu.edu.cn

Table of Contents

Figure S1. ^{1}H NMR spectrum of PTSA S3

Figure S2. ^{1}H NMR spectrum of PcSA S3

Figure S3. HRMS spectrum of PTSA S4

Figure S4. HRMS spectrum of PcSA S4

Figure S5. ^{1}H NMR spectrum of PTOA S5

Figure S6. ^{1}H NMR spectrum of PcOA S5

Figure S7. HRMS spectrum of PTOA S6

Figure S8. HRMS spectrum of PcOA S6

Figure S9. Particle size and TEM images of PcSA in water S7

Figure S10. $O_2^{\bullet-}$ generation by PcSA, PcOA and MB in water S7

Figure S11. $^{1}O_2$ generation by PcSA, PcOA and MB in water S8

Figure S12. Photothermal activity of PcSA and PcOA in water S8

Figure S13. $O_2^{\bullet-}$ and $^{1}O_2$ generation of PcSA@Lip in water S9

Figure S14. Cytotoxicity and cellular uptake S9

Table S1. Photo-physical/photo-chemical and the Log $P_{w/o}$ data of zinc(II) phthalocyanines S9

Table S2. The IC_{50} value of HepG2 cells upon light irradiation S10

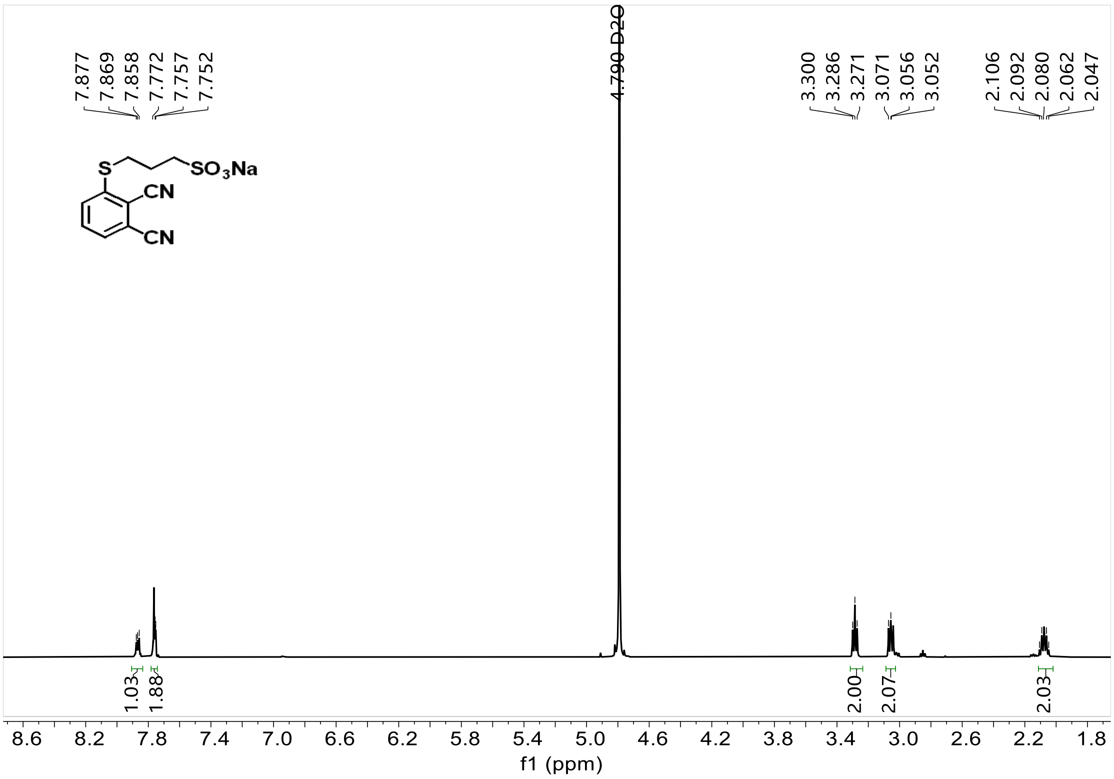

Figure S1. ^{1}H NMR spectrum of PTSA in D_2O.

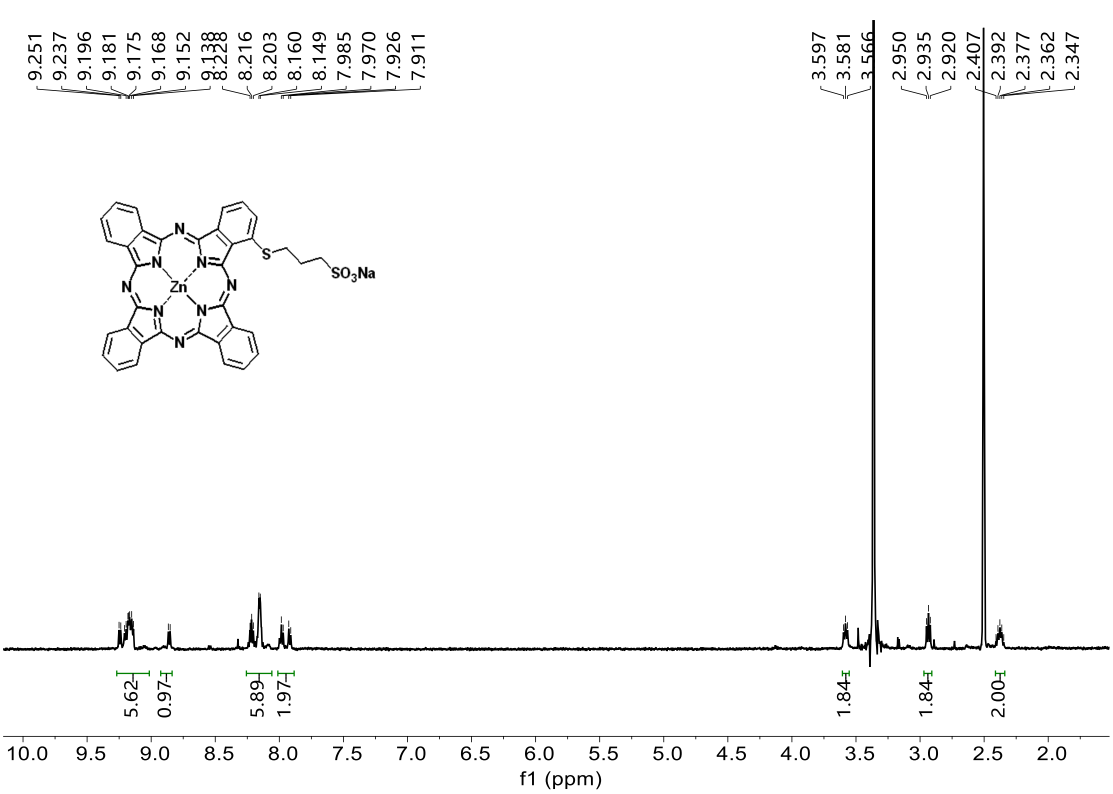

Figure S2. ^{1}H NMR spectrum of PcSA in DMSO-d.

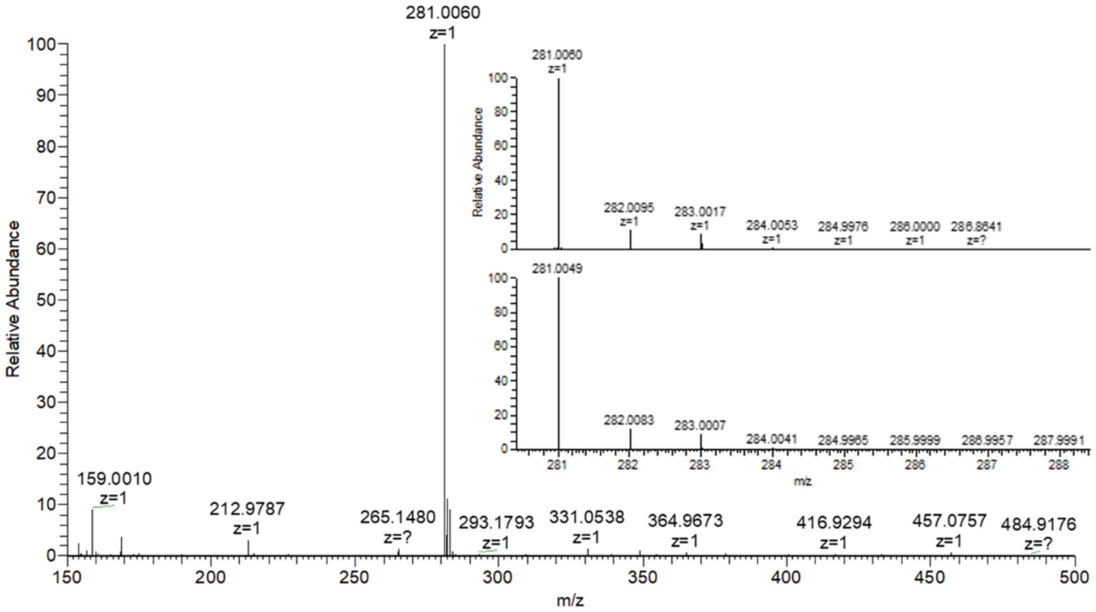

Figure S3. HRMS spectrum of PTSA. The inset shows the enlarged isotopic envelop for the [M-Na]$^-$ species.

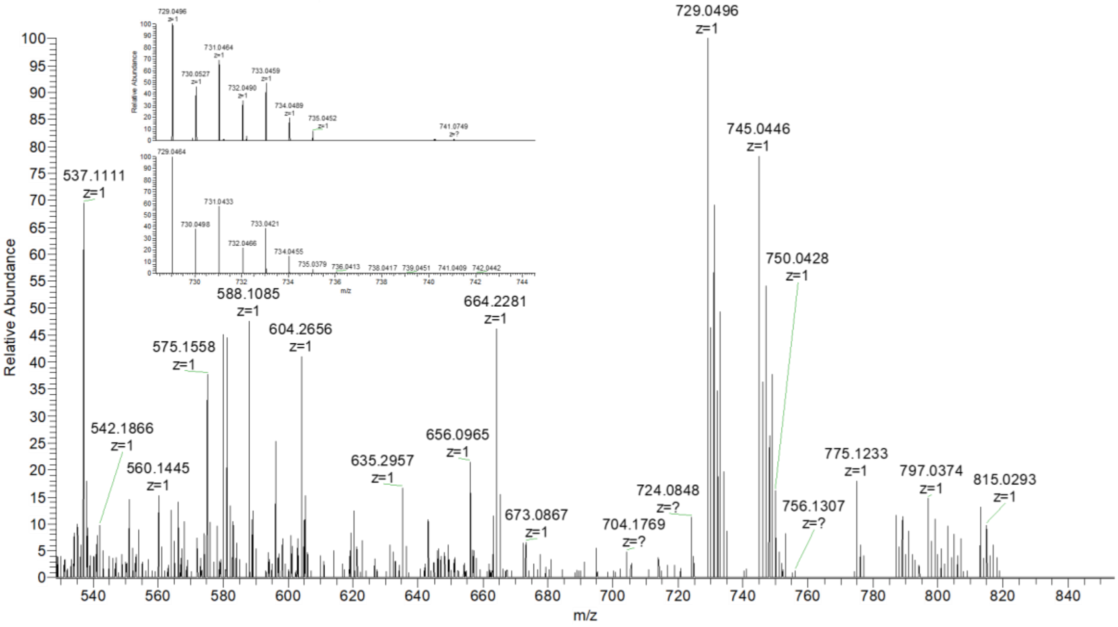

Figure S4. HRMS spectrum of PcSA. The inset shows the enlarged isotopic envelop for the [M-Na]$^-$ species.

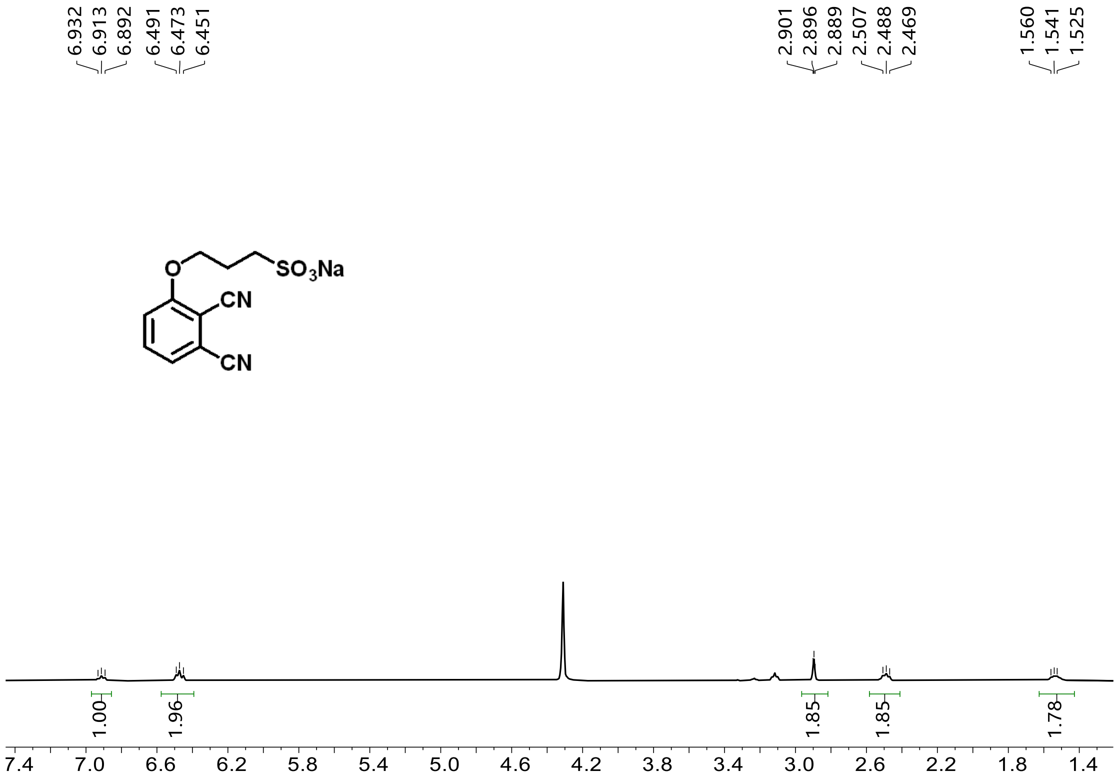

Figure S5. ^{1}H NMR spectrum of PTOA in D_2O.

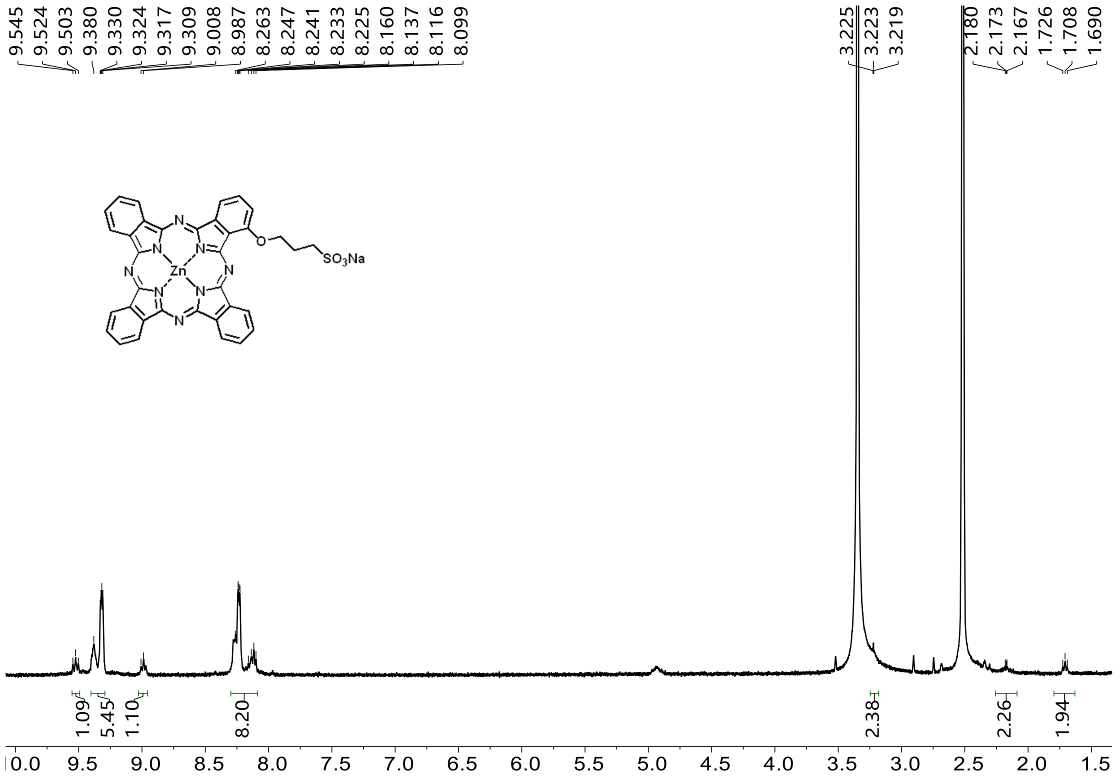

Figure S6. ^{1}H NMR spectrum of PcOA in DMSO-d.

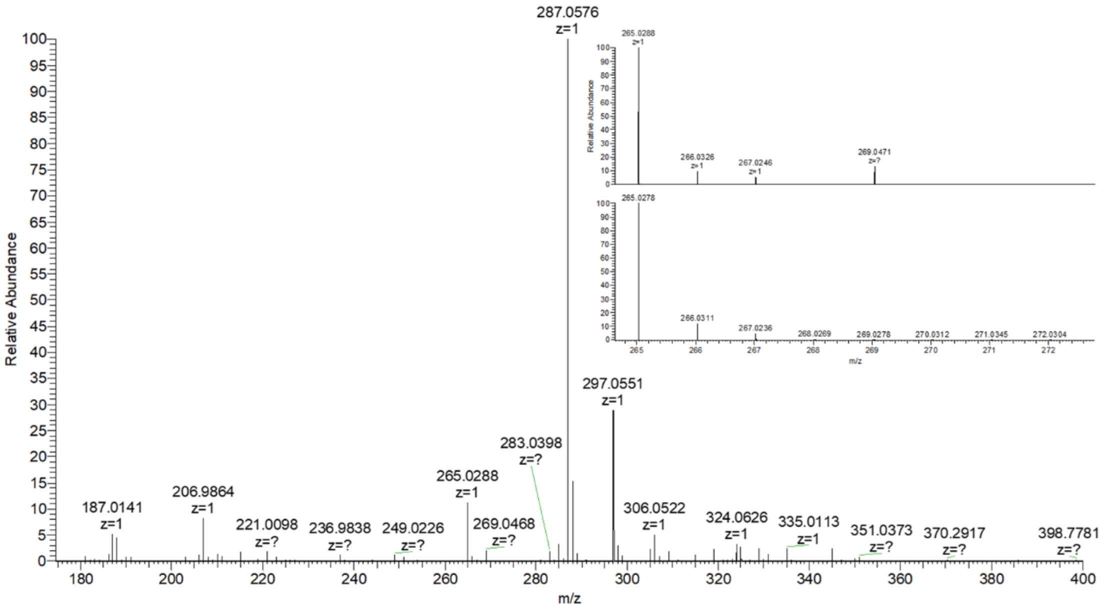

Figure S7. HRMS spectrum of PTOA. The inset shows the enlarged isotopic envelop for the [M-Na]$^-$ species.

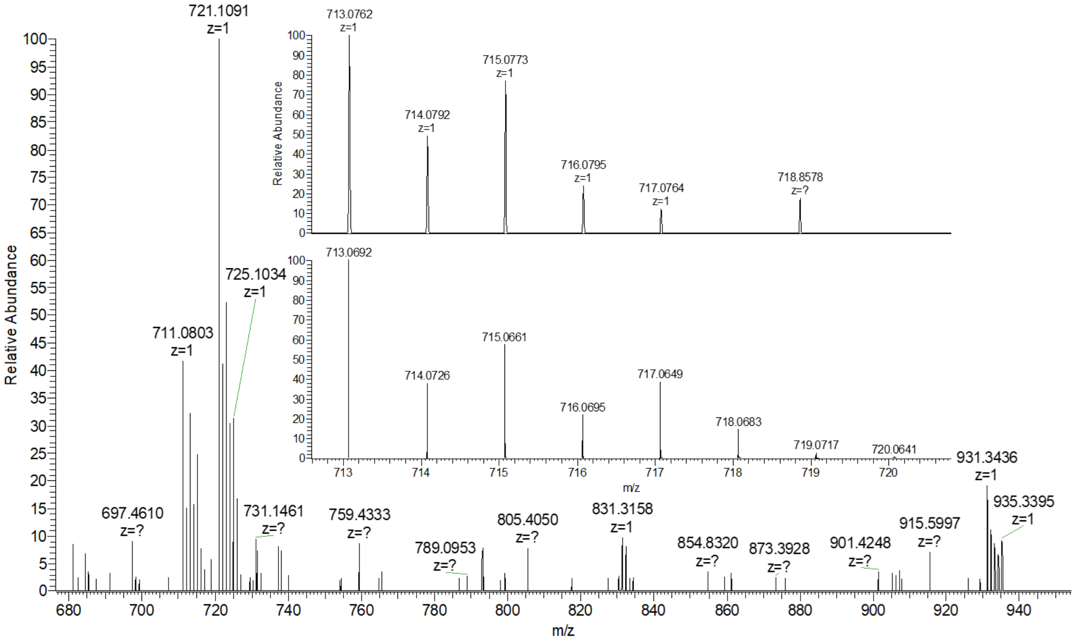

Figure S8. HRMS spectrum of PcOA. The inset shows the enlarged isotopic envelop for the [M-Na]$^-$ species.

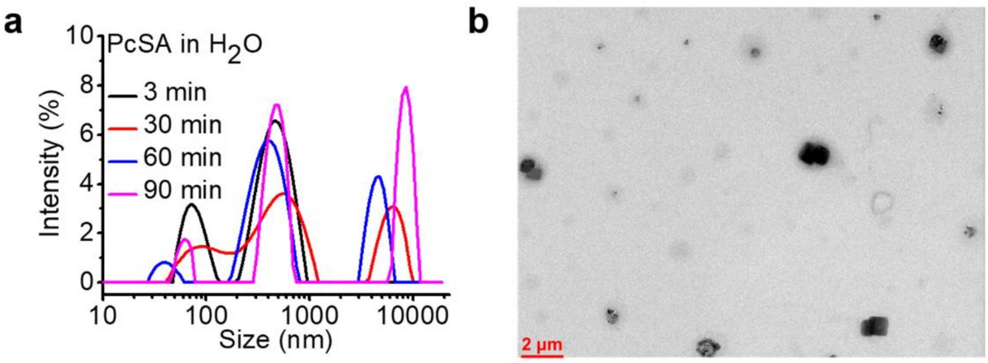

Figure S9. (a) Size distribution of PcSA (4 μM) in water detected by DLS. (b) TEM images of PcSA (2 μM) in water.

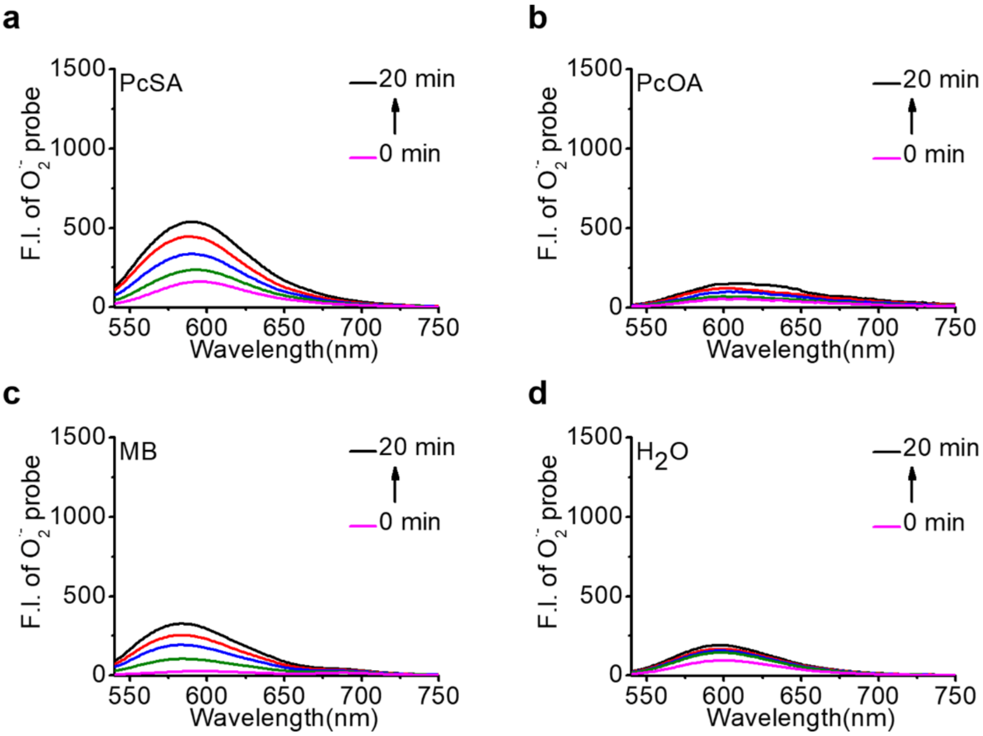

Figure S10. The $O_2^{\bullet-}$ generation of (a) PcSA, (b)PcOA, (c) MB (all at 4 μM) and (d) blank in water using DHE as probe, λ_{ex} = 510 nm. F.I., fluorescence intensity.

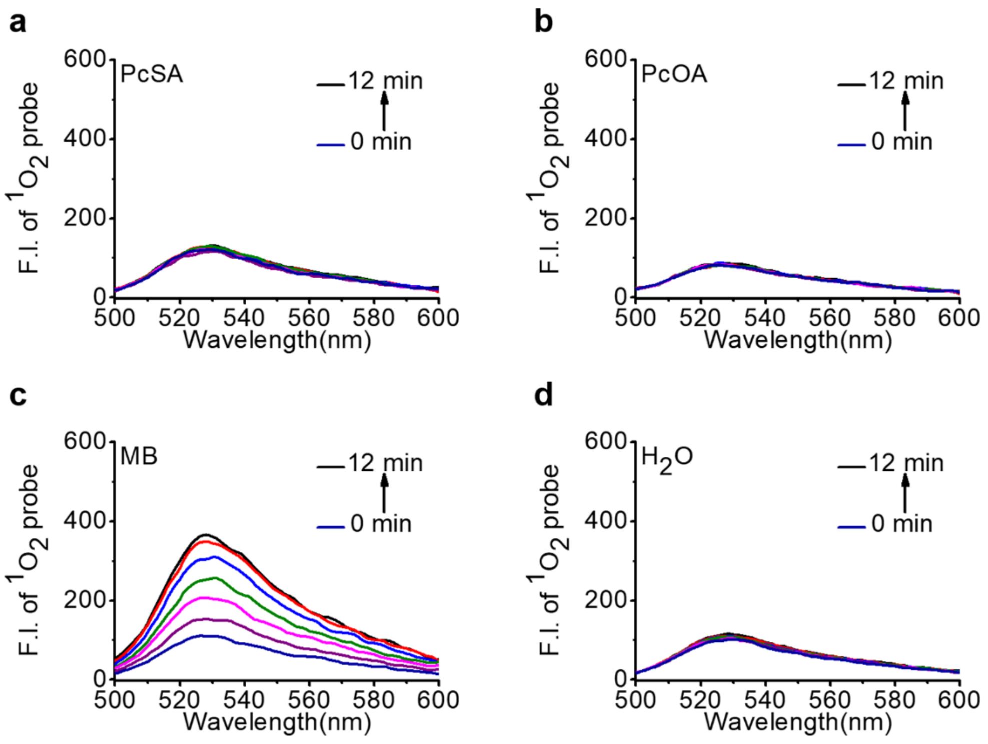

Figure S11. The $^1O_2$ generation of (a) PcSA, (b)PcOA, (c) MB (all at 4 μM) and (d) blank in water using SOSG as probe, λ_{ex} = 488 nm. F.I., fluorescence intensity.

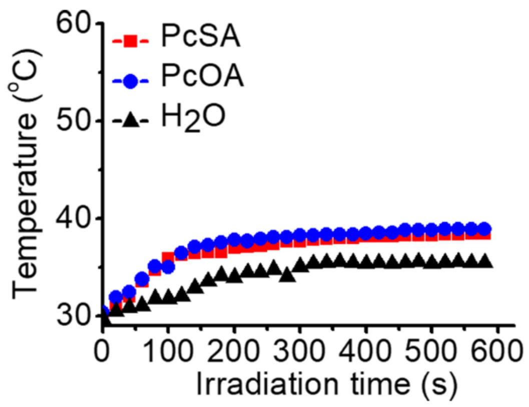

Figure S12. Time dependent temperature changes of PcSA, PcOA (both at 10 μM) in water with laser illumination (680 nm) for 10 min (0.5 W/cm^2). Water was used as control.

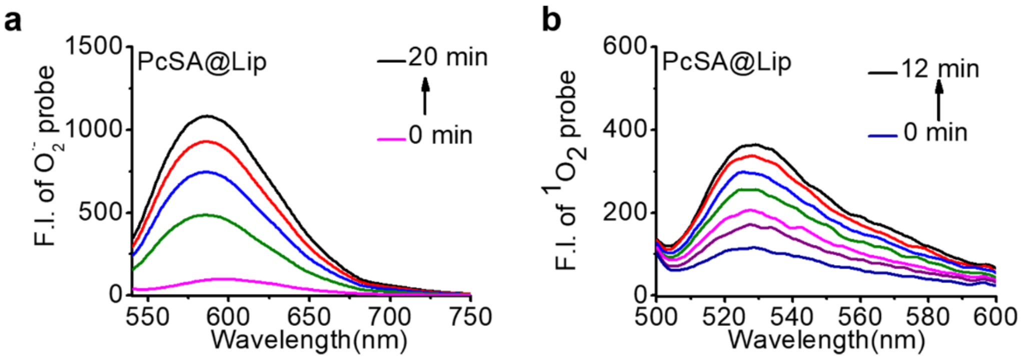

Figure S13. The generation of (a) $O_2^{\bullet-}$ and (b) $^1O_2$ by PcSA@Lip ([PcSA] = 4 μM) in water. F.I., fluorescence intensity.

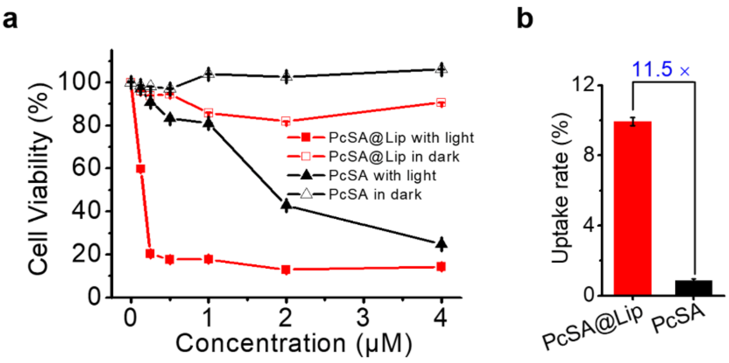

Figure S14. (a) Cytotoxicity of PcSA@Lip and PcSA on HepG2 cells in the presence and absence of light (λ > 610 nm, 27 J/cm^2). (b) Cellular uptake of PcSA@Lip and PcSA ([PcSA] = 4 μM), respectively. Data were showed as the mean ± standard deviation, n = 3.

Table S1. Photo-physical/photo-chemical data of zinc(II) phthalocyanines in DMF, and the PBS/octanol partition coefficient ($P_{w/o}$) data for zinc(II) phthalocyanins.

Compounds	λ_{max}/nm	λ_{em}/nm [a]	Φ_F [b]	Φ_Δ [c]	Log $P_{w/o}$
PcSA	680	688	0.22	0.76	-0.97
PcOA	671	677	0.11	0.65	-0.94
ZnPc	668	675	-	-	2.39

[a] Excited at 610 nm. [b,c] Using ZnPc in DMF as the reference (Φ_F = 0.28, Φ_Δ = 0.56).

Table S2. The IC_{50} value of HepG2 cells upon light irradiation.

PSs	IC_{50} against HepG2 cells
PcSA@Lip	0.16 ± 0.04 μM
PcSA	1.81 ± 0.12 μM
